# How heterogeneous is the dengue transmission profile in Brazil? A study in six Brazilian states

**DOI:** 10.1371/journal.pntd.0010746

**Published:** 2022-09-12

**Authors:** Iasmim Ferreira de Almeida, Raquel Martins Lana, Cláudia Torres Codeço

**Affiliations:** 1 Department of Epidemiology, Escola Nacional de Saúde Pública Sergio Arouca, ENSP/ FIOCRUZ, Rio de Janeiro, Brazil; 2 Programa de Computação Científica, PROCC/FIOCRUZ, Rio de Janeiro, Brazil; 3 Barcelona Supercomputing Center, BSC, Barcelona, Spain; Louisiana State University, UNITED STATES

## Abstract

Dengue is a vector-borne disease present in most tropical countries, infecting an average of 50 to 100 million people per year. Socioeconomic, demographic, and environmental factors directly influence the transmission cycle of the dengue virus (DENV). In Brazil, these factors vary between regions producing different profiles of dengue transmission and challenging the epidemiological surveillance of the disease. In this article, we aimed at classifying the profiles of dengue transmission in 1,823 Brazilian municipalities, covering different climates, from 2010 to 2019. Time series data of dengue cases were obtained from six states: Ceará and Maranhão in the semiarid Northeast, Minas Gerais in the countryside, Espírito Santo and Rio de Janeiro in the tropical Atlantic coast, and Paraná in the subtropical region. To describe the time series, we proposed a set of epi-features of the magnitude and duration of the dengue epidemic cycles, totaling 13 indicators. Using these epi-features as inputs, a multivariate cluster algorithm was employed to classify the municipalities according to their dengue transmission profile. Municipalities were classified into four distinct dengue transmission profiles: persistent transmission (7.8%), epidemic (21.3%), episodic/epidemic (43.2%), and episodic transmission (27.6%). Different profiles were associated with the municipality’s population size and climate. Municipalities with higher incidence and larger populations tended to be classified as persistent transmission, suggesting the existence of critical community size. This association, however, varies depending on the state, indicating the importance of other factors. The proposed classification is useful for developing more specific and precise surveillance protocols for regions with different dengue transmission profiles, as well as more precise public policies for dengue prevention.

## Author summary

Dengue is one of the fastest-growing vector-borne diseases in the world. Currently, vaccines are experimental and are not very effective, so prevention depends on the control of the mosquito *Aedes aegypti*. Health promotion campaigns aimed at encouraging people to reduce mosquito breeding sites have limited effect. In addition, the heterogeneity of the territories that have dengue becomes a major challenge for the epidemiological surveillance of the disease. Brazil has a territory of continental size, and single standardized surveillance is not very effective for monitoring this arbovirus. Classifying types of dengue dynamics based on features of the epidemiological cycle in each location has the potential to increase the precision of surveillance and control strategies. In our study, we were able to classify areas according to different dengue transmission profiles, ranging from episodic to persistent transmission. These results can provide tools to guide actions aimed at achieving the World Health Organization’s goals of eliminating neglected tropical diseases in countries that have the virus.

## Introduction

Dengue is a disease of great public health concern worldwide, mainly in growing urban centers of tropical regions [1–3]. Brazil is one of the tropical countries with the highest dengue disease burden [4,5]. From 2003 to 2019, a total of 11,137,664 dengue cases were reported, with an average annual mortality rate of 3.05/100,000 inhabitants. There were five epidemic years in this period, the highest one in 2019, when 1,544,987 cases were reported [6]. Dengue emerged in Brazil in the 1980s, quickly spreading to the country´s urban areas. In the last decade, dengue has spread to more rural areas [7] and higher latitudes [8].

Brazil’s continental size and socioeconomic, demographic, climatic, and environmental heterogeneity affect the local temporal patterns of dengue transmission, contributing to disease maintenance at endemic levels in some areas, alternating with areas characterized by epidemic periods throughout almost the entire Brazilian territory [9–11]. The *Aedes aegypti* mosquito is the main dengue vector, while *Aedes albopictus* is considered a potential vector in rural and semi-urban [12]. The abundance of mosquito breeding sites is directly related to lack of urban infrastructure, water services, precarious housing conditions in areas with high social vulnerability, and high human density [13,14]. Moreover, the climate has a strong effect on the biological cycle of *Ae*. *aegypti*. Reproduction rate, mosquito abundance, and longevity are important components of the vectorial capacity and are all dependent on adequate temperature and humidity [15–17]. Low relative humidity impairs the longevity of the mosquito and its vectorial capacity, while low temperature inhibits mosquito and virus development. As a result, dengue transmission is seasonal, concentrated in the warm and humid months [18–20].

In a context of heterogeneous population density and different climate regimes, it is expected that dengue transmission will not behave similarly in all places. Less populated municipalities in drier or cooler climates will experience less dengue outbreaks than municipalities with large urban populations in wet and humid climates. Discriminating these different profiles is important for many reasons. First, from an academic perspective, the existence of a critical community size for dengue, above which dengue transmission is more persistent has been debated [21–24]. Whether this phenomenon can be attributed to local immunity or to environmental conditions is an open question. Second, from a practical perspective, the classification of municipalities into transmission profiles can assist the development of more precise monitoring and public health strategies. For example, a control diagram is a typical method used to detect epidemic years in Brazil [25] that is only applicable in places with persistent or seasonal transmission. In areas with the occasional transmission, a Poisson model would be more adequate [26]. The study thus aimed to characterize the dengue transmission profiles in 1,823 municipalities located in six Brazilian states with different climates and latitudes, seeking to identify those with persistent, episodic, and epidemic. Municipalities with persistent dengue transmission are expected to present relatively high dengue incidence, with rare periods with low or no cases. Those characterized by episodic transmission, on the other hand, should present lower and more irregular case counts, with short periods of disease transmission, due to susceptible depletion or unfavorable environmental conditions. Municipalities with an epidemic dengue profile, at last, are expected to present a strong seasonal signal with high incidence periods interspersed with periods of low incidence [27,28].

## Methods

### Data

The disease data consist of dengue cases reported from residents of 1,823 municipalities located in six Brazilian states, from September 2010 to September 2020. The source is the Infodengue platform (https://info.dengue.mat.br/) an arbovirus alert system that harmonizes official dengue notification data from the Brazilian Notifiable Diseases Information System (SINAN), with climate data from meteorological stations at Brazilian airports [29]. Data from SINAN (http://portalsinan.saude.gov.br/) are public and aggregated by week and municipality and, according to current Brazilian laws, there is no need for additional approval from the ethics committee.

For the analysis, all reported cases were included, regardless of the type of confirmation, except those later discarded by the Health System. In Brazil, the laboratory confirmation rate is relatively low and varies significantly between states and between years. Since dengue is a well-known disease in Brazil and prevalent, we considered that the notified cases were the best available estimate of the true cases. To calculate annual dengue incidence, the population size of each municipality for the year 2019 was obtained from a projection calculated by the Brazilian Institute of Geography and Statistics [30].

**Fig 1:**
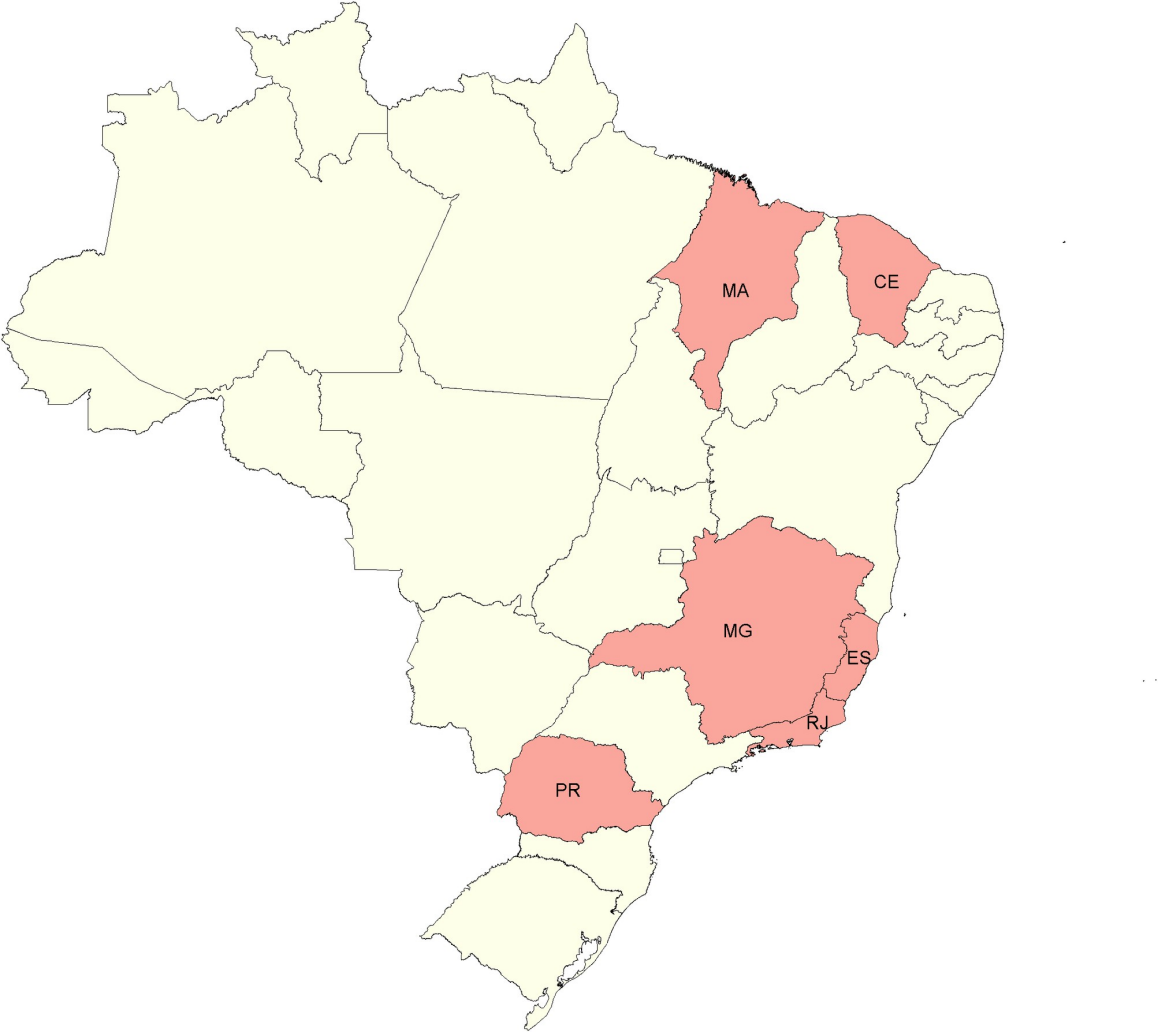
Map of Brazil showing the six states analyzed. The states are presented from north to south: Maranhão (MA), Ceará (CE), Espírito Santo (ES), Minas Gerais (MG), Rio de Janeiro (RJ), and Paraná (PR) and the series from 2010 to 2019. Service Layer Credits: Sources: https://www.ibge.gov.br/geociencias/organizacao-do-territorio/malhas-territoriais/15774-malhas.html?=&t=downloads.

The six states represent different climates, biomes, and population densities. Ceará and Maranhão are states located in the Northeast region, the former being characterized by a mixture of areas with tropical climate with dry summers and areas dominated by a dry semiarid climate, while the latter state is characterized by a more humid climate, in the transition between the Savanna and the Amazonian biomes. Both states have small to medium-sized municipalities, the largest ones being the capitals, with 2,686,612 and 1,108,975 inhabitants, respectively. Minas Gerais, Espírito Santo, and Rio de Janeiro are neighboring states located in the populous and more urbanized Southeast region. Minas Gerais is a large state in the countryside, with a climate varying from tropical with dry summers to humid subtropical with temperate summers and dry winters in the mountainous area. Espírito Santo and Rio de Janeiro are smaller states along the coast, characterized by tropical climates with humid summers and relatively drier winters. Paraná is located in the South of Brazil, in the subtropical zone, characterized by warm summers in the western and eastern parts and cooler summers in the central area [30,31]. Fig 1 shows the location of the states and the time series of dengue cases from 2010 to 2019.

### Epidemic features (Epi-features)

To describe the time series of dengue transmission in each municipality, we developed a set of summary descriptors inspired by the concept of epi-features proposed by Tabataba *et al*. (2017) [32] for respiratory diseases. Epi-features are features (motifs) used to describe the shape of an epidemiological time series. These motifs are chosen according to the problem at hand. Feature extraction is a method classically applied to signal processing problems [33] and has been recently revisited in the context of forecast models for transmissible diseases [32,34,35]. Some of these features, such as the peak and duration of the transmission season, are broadly used when comparing transmission suitability among different places [36,37]. Besides these, we also considered features that are specific for the study objectives, that is, features related to the expected differences between episodic, epidemic and endemic time series (Table 1). Dcmax or Dsmax are features describing the length of period with/without records of dengue cases, which is directly related to the sustainability of transmission [8,38]. The seasonal-to-trend ratio (ST) is also a standard metric in signal processing analysis for measuring the importance of the seasonal transmission compared to the baseline transmission [39]. In summary, all of these are the features proposed to describe important dengue time series signatures that can be related to the literature and were used to establish dengue transmission profiles.

**Table 1:** Epi-features are proposed to characterize the time series of dengue cases.

Epi-features	Abbreviation	Definition
**Peak amplitude**	**Xp**	Maximum value of incident cases per week in an epidemiological year. Unit of measure: cases. Example: **[8, 15, 20**, 0, 0, 0, 0, **5, 9, 12]** has Xp = 20.
**Peak time**	**Tp**	Week in which peak of case incidence is reached (Xp). Unit of measure: epidemiological week.
**Length of the period with cases (transmission)**	**Dc3**	Frequency of at least three consecutive weeks with five or more dengue cases during an epidemiological year. Unit of measure: dimensionless (count). Example: **[8, 15, 20**, 0, 0, 0, 0, **5, 9, 12]** has Dc3 = 2.
	**Dc6**	Frequency of at least six consecutive weeks with five or more dengue cases during an epidemiological year. Unit of measure: dimensionless (count). Example: **[5, 8, 9, 10, 8, 6**, 0, 0, 0, 12] has Dc6 = 1.
	**Dcmax**	Maximum number of consecutive weeks within one epidemiological year with five or more dengue cases. Unit of measure: week.
	**Dcmed**	Median length of consecutive weeks with five or more dengue cases. Unit of measure: week.
	**Dci**	Frequency of isolated weeks with five or more dengue cases during the epidemiological year. Unit of measure: count. Example = [0, **16**, 0, 0, 8, 3, 0, **10**, 0, 2, 0]. Dci = 2
**Length of case-free period (transmission)**	**Ds3**	Frequency of at least three consecutive weeks with no record of dengue cases during the epidemiological year. Unit of measure: dimensionless (count). Example: [2, **0, 0, 0,** 3, 5, **0, 0, 0, 0**, 2] has Ds3 = 2.
	**Ds6**	Frequency of at least six consecutive weeks with no record of dengue cases during the epidemiological year. Unit of measure: dimensionless (count). Example: [3, **0, 0, 0, 0, 0, 0,** 3, 5, **0, 0, 0,** 2] has Ds6 = 1.
	**Dsmax**	Maximum period of consecutive weeks within an epidemiological year with no record of dengue cases. Unit of measure: week.
	**Dsmed**	Median length of a one-year epidemiological period with no record of dengue cases. Unit of measure: week.
**Proportion of positive weeks**	**P+**	Frequency of epidemiological weeks with five or more cases per year. Unit of measure: dimensionless (proportion).
**Trend/seasonality ratio**	**ST**	Ratio between the average trend and the average seasonality component obtained from the multiplicative decomposition of the time series. Unit of measure: dimensionless.

Here, we adapted and proposed new epi-features to describe the transmission pattern of dengue during an epidemiological year, including measures such as the magnitude of cases, the length of periods with and without notified cases, and measures of seasonality and trend (Table 1). Before computing the epi-features, the dengue time series of each municipality was cut in 10 epidemiological years. The epidemiological year of dengue in Brazil is defined as the period from the last week of September of one year to the last week of September of the following year, encompassing the whole period of dengue transmission, whose peak tends to occur in February-May [7,8].

### Multivariate cluster analysis

Multivariate cluster analysis was performed to define groups of municipalities with similar epi-features First, a correlation matrix was calculated for all proposed epi-features. Highly correlated epi-features (>0.80) were analyzed and three were removed from subsequent analysis to reduce collinearity. Between Dsmax and Dsmed, we opted to keep Dsmax although similar results are obtained using Dsmed. Dsmax is a good descriptor of longest periods without cases. Between P and Dcmax, we kept Dcmax, accounting for the longest periods with cases. Although not highly correlated, we dropped Dsmed from the analysis, since its counterpart Dcmed was removed, as well. The correlation matrix of the final set of variables is shown in (S1 Fig). Second, we computed the average for each epi-feature over the 10 years for each municipality to obtain a vector of typical epi-features per municipality. We also considered computing the median instead of the average, but the results were similar (not shown). Epi-features were standardized, with the aim of normalizing the data to establish a common scale, so that there is no distortion of values when there are larger ranges.

The clustering algorithm of choice was PAM (partitioning around medoids) because of its robustness, low sensitivity to outliers and its ability to preselect the number of clusters [40,41]. The elbow method was applied to give insight into the optimal number of cluster groups [42], while the silhouette method was used to assess the quality of the resulting partitions produced by PAM [41–43]. To guide the interpretation of the cluster analysis, summary statistics of the epi-features stratified by cluster were calculated, and maps of the classification were produced. The population distribution per municipality and the average dengue incidence during the study period were also plotted in maps to aid the interpretation. All analyses were performed using R Core Team, version 4.0.2 (2020).

## Results

### Distribution of population and dengue cases

**Fig 2:**
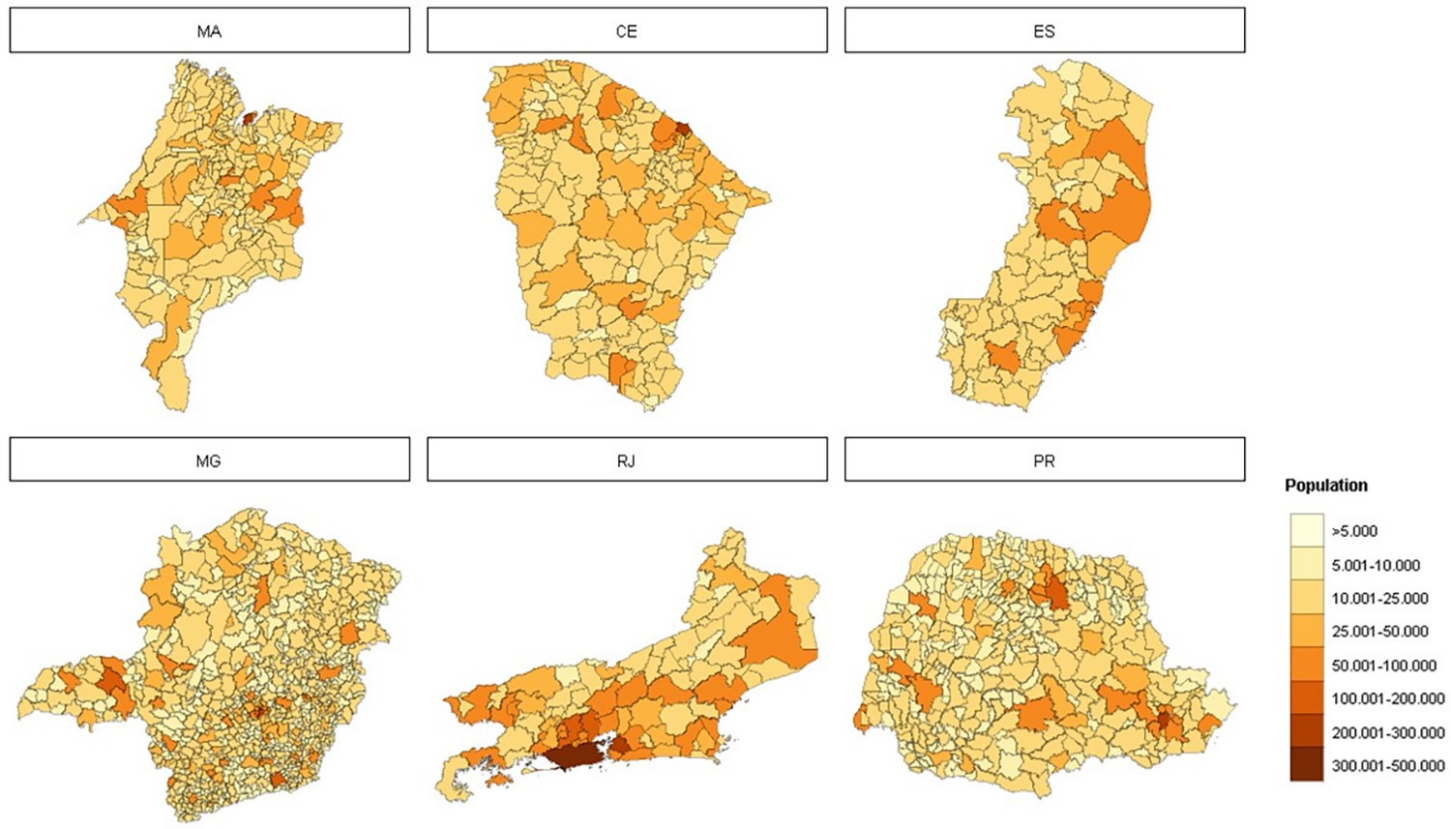
Population distribution in six states of Brazil, 2019. The states are shown from north to south: Maranhão (MA), Ceará (CE), Espírito Santo (ES), Minas Gerais (MG), Rio de Janeiro (RJ), and Paraná (PR). Service Layer Credits: Sources: https://www.ibge.gov.br/geociencias/organizacao-do-territorio/malhas-territoriais/15774-malhas.html?=&t=downloads.

The six states under investigation account for 29.5% (62,268,835 inhabitants) of the Brazilian population. Fig 2 shows the population distribution within the studied states. Thirty percent of the population live in Minas Gerais, the largest state in total area and number of municipalities. The second most populous state is Rio de Janeiro (24.6%), followed by Paraná (16.3%), Ceará (13.0%), Maranhão (10.1%), and Espírito Santo (5.7%). Among all the municipalities, 68.3% have fewer than 20,000 inhabitants. In Minas Gerais and Paraná, 77–78% of the municipalities are within this low population range, while in Maranhão, Ceará, and Espírito Santo they correspond to 50% of the municipalities. In Rio de Janeiro, only 28% of the municipalities are below this population threshold. On the other extreme, there are 19 municipalities with more than 500,000 inhabitants, including all the state capitals. There are four such populous cities in Minas Gerais and seven in Rio de Janeiro [30].

**Fig 3:**
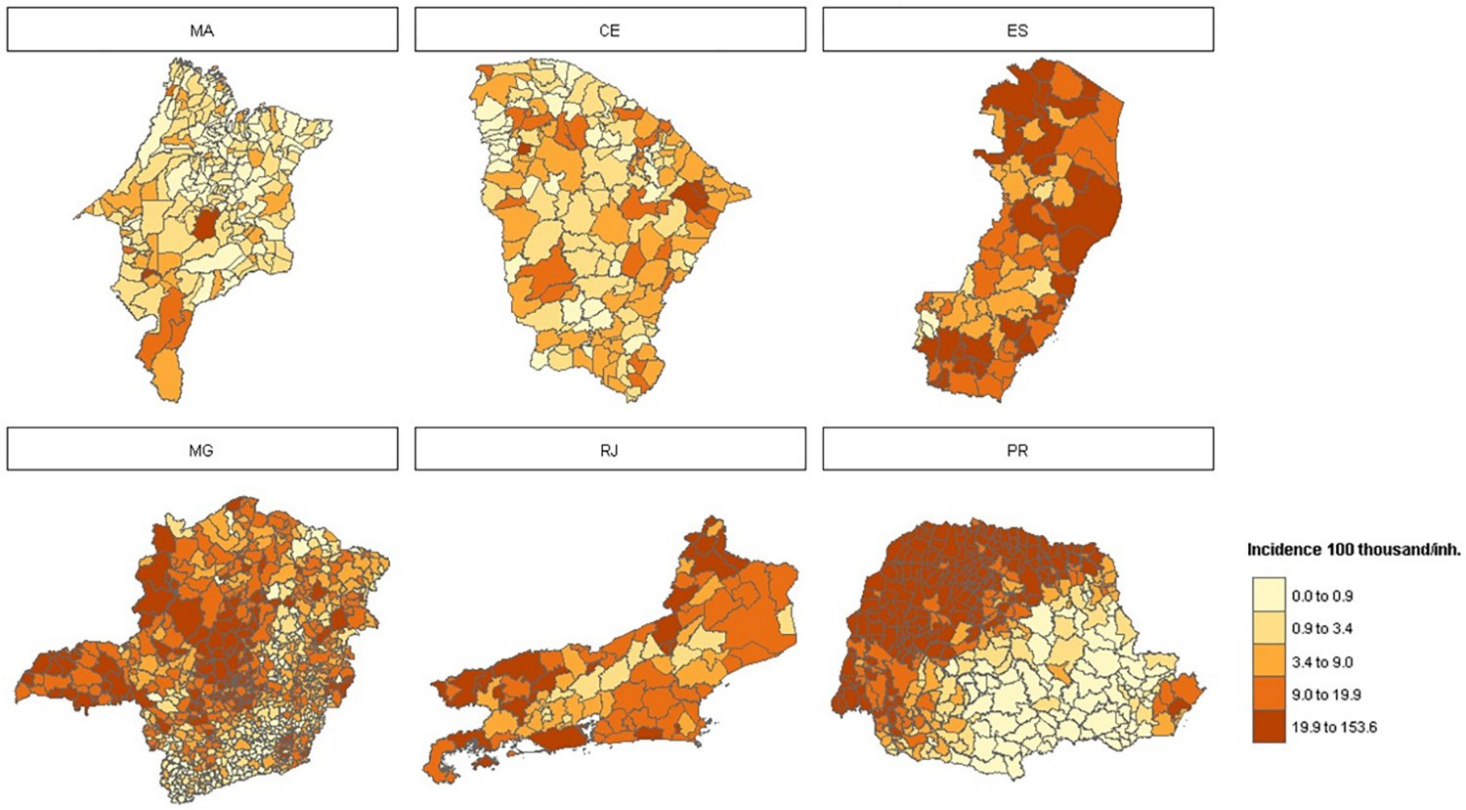
Maps of mean dengue incidence per municipality in the period 2010 to 2019. The states are shown from north to south: Maranhão (MA), Ceará (CE), Espírito Santo (ES), Minas Gerais (MG), Rio de Janeiro (RJ), and Paraná (PR). Service Layer Credits: Sources: https://www.ibge.gov.br/geociencias/organizacao-do-territorio/malhas-territoriais/15774-malhas.html?=&t=downloads.

During the study period, an average of 2,568 cases were reported per epidemiological year per municipality, ranging from 0 to 577,674 cases. Fig 3 shows the mean dengue incidence (100,000 /inhab.) by municipality. Maranhão (MA) has the lowest incidence rates with cases concentrated in the state capital, São Luis, and in the westernmost municipalities. Ceará showed a higher heterogeneity in dengue incidence, with neighboring municipalities showing contrasting incidences. Espírito Santo and Rio de Janeiro presented high dengue incidence in almost all municipalities, while in Minas Gerais the highest incidence was found in the western and eastern parts of the state. Paraná showed a clear spatial pattern, with high incidence in the west and a few municipalities in the east. In absolute terms, the average number of cases per epidemiological year was 2,337.5 in Ceará, 5,156.2 in Espírito Santo, 420.5 in Maranhão, 2,477.3 in Minas Gerais, 2,698.5 in Paraná, and 10,580.5 in Rio de Janeiro.

### Epi-features

**Fig 4:**
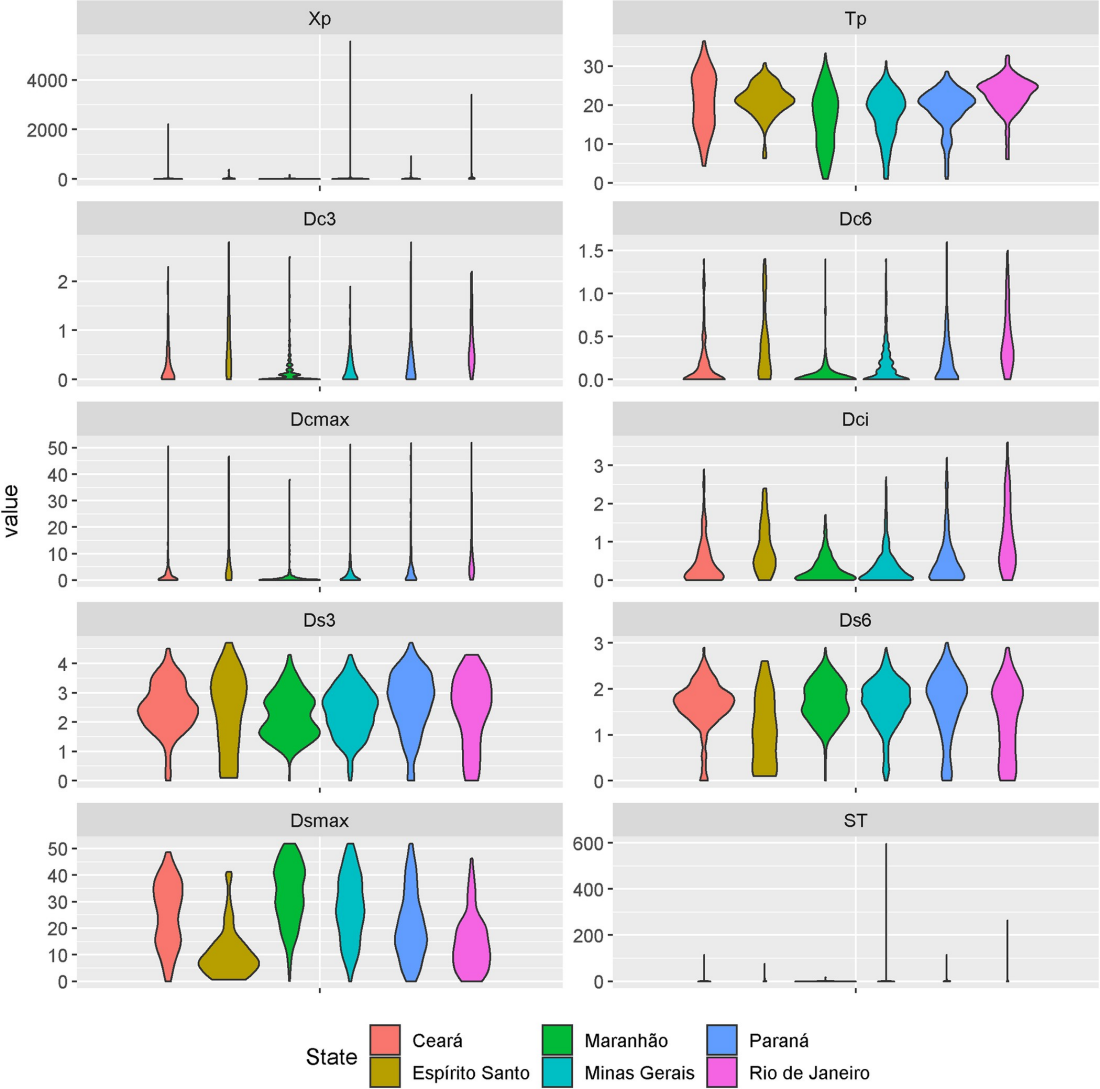
Distribution of (untransformed) epi-features summarizing the dengue time series in six states of Brazil. From north to south: Maranhão (MA), Ceará (CE), Espírito Santo (ES), Minas Gerais (MG), Rio de Janeiro (RJ), and Paraná (PR).

Distribution of epi-features per state is shown as violin plots in Fig 4.

The magnitude of the epidemic peak (Xp) varied widely between municipalities, with 95.4% showing Xp fewer than 100 cases. At the other extreme, there were municipalities with very high epidemic peaks, for example, Belo Horizonte (MG), with Xp = 5,563 cases. In most municipalities (1,150 or 63.1%), the peak occurred between weeks Tp = 15 and Tp = 25, corresponding to the summer months in Brazil. In MA and CE, the northeastern states, the week of the epidemic peak varies more and can occur later in the year, ranging from Tp = 1 to 34 in MA and Tp = 5 to 36 in CE. The epi-feature Dc3 ranged between 0 and 2.8. Dc3 equal to 0 means the absence of any 3-week period with continuous reporting of dengue cases throughout the entire decade. There are 604 municipalities with Dc3 = 0, mainly in Minas Gerais (299 or 49.5%) and Maranhão (125 or 20.7%). All states had at least one municipality with Dc3 > 1, which indicates intermittent transmission, that is, transmission periods interspersed by weeks with 0 cases (133 or 7.9%). The epi-feature Dc6 measures the occurrence of longer transmission periods, of at least six weeks or two-generation cycles. These were more frequent in Rio de Janeiro and Espírito Santo. Dcmax measures the duration of the longest period with cases during an epidemiological year. It ranged from 0 to 52 weeks (mean = 3.4 weeks). The longest periods of continuous transmission occurred in the municipalities of Rio de Janeiro (RJ) and Londrina (PR), while the shortest periods were in the state of Maranhão. Dci measures the presence of isolated dengue detection events (a positive week preceded and followed by negative weeks). Dci ranged from 0 to 3.6 (mean = 0.46), with 39 municipalities (2.13%) presenting mean Dci = 1, that is, only one isolated week per year with five or more dengue cases (Dci). Such events were frequent in all states except Rio de Janeiro and Espírito Santo.

The epi-features Ds3, Ds6, and Dsmax characterize periods with no dengue cases. Ds3 varied from 0 to 4.7 (mean = 2.4), while Ds6 varied from 0 to 3 (median = 2.6). Events of six weeks or more without cases occurred with an average frequency of once a year (Ds6). It ranged from 0 to 1.3–1.6 across all the states and was more frequent in Maranhão and Ceará in the Northeast and Minas Gerais in the Southeast. The distributions of Ds3 and Ds6 were similar in CE and MA, ranging mostly between values Ds3 = 1 to 3 and Ds6 = 1 to 2.5. The other states presented higher variability, particularly RJ and ES. The maximum period without cases, Dsmax, varied from 0 to 52 weeks (mean = 25.17). There were 28 municipalities without any record of dengue during the whole year (Dsmax = 52), including Cajapió (MA), Bias Fortes (MG) and Agudos do Sul (PR). All are small municipalities with low population density. The latter two have cold climates, with mean temperatures from 10 to 15°C.

Finally, ST is an indicator of the trend-to-seasonality ratio. Low values imply a strong seasonal signal relative to the historical trend of the municipality, while high values indicate the opposite. ST varied from 0 to 597, with values ​​concentrated between 0 and 10. Belo Horizonte (MG) is an example of high trend-to-seasonality ratio, where the seasonal component was 2.76 and the trend was 316.9, resulting in ST = 597.04. Bacuritiba (MA), on the other hand, is an example of strong seasonality compared to the historical trend, with the seasonal component = 52 and trend = 0.0019, resulting in ST = 0.001923.

### Classification and interpretation of transmission profiles

**Fig 5:**
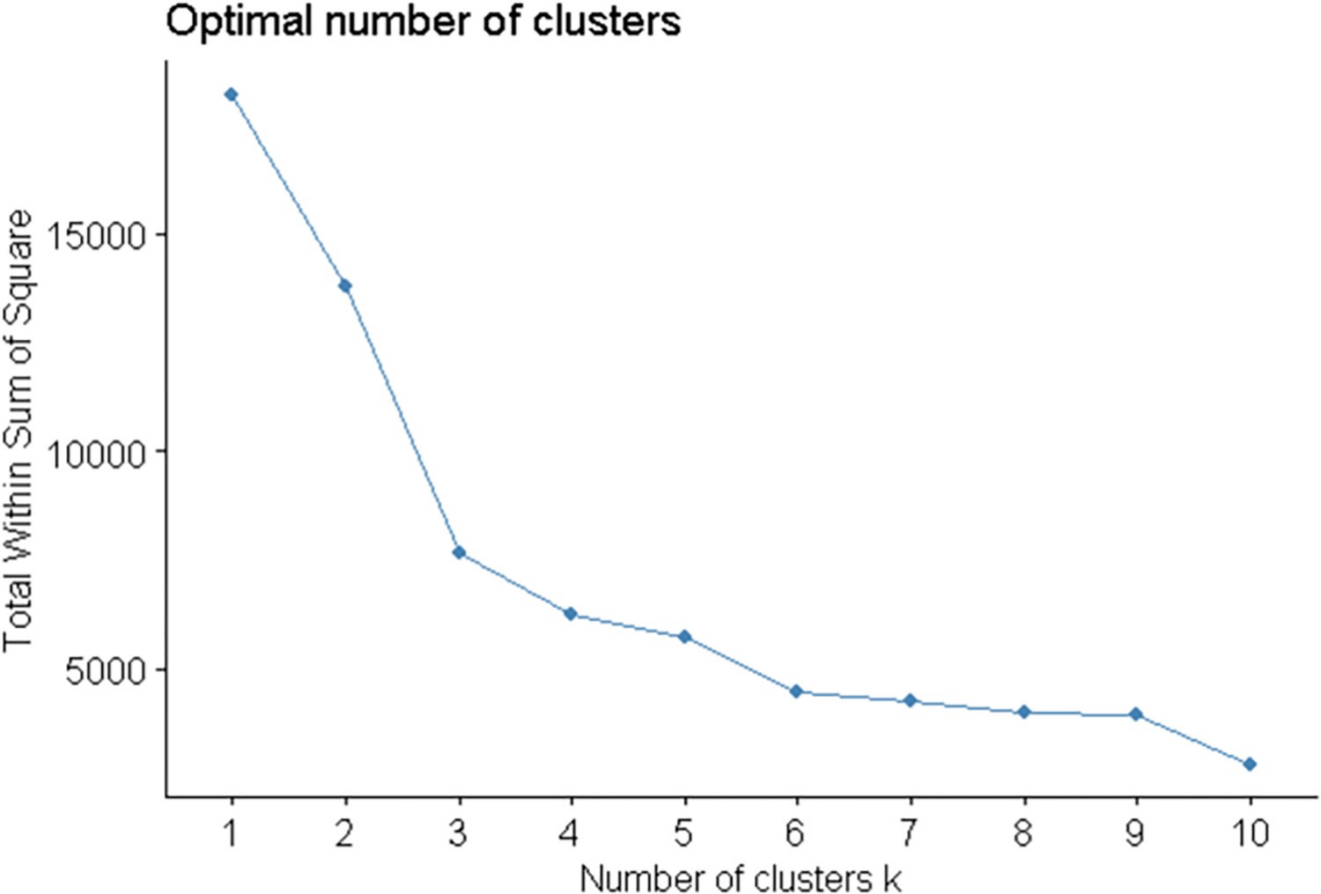
Elbow method chart applied to data from municipalities in the six states analyzed.

**Fig 6:**
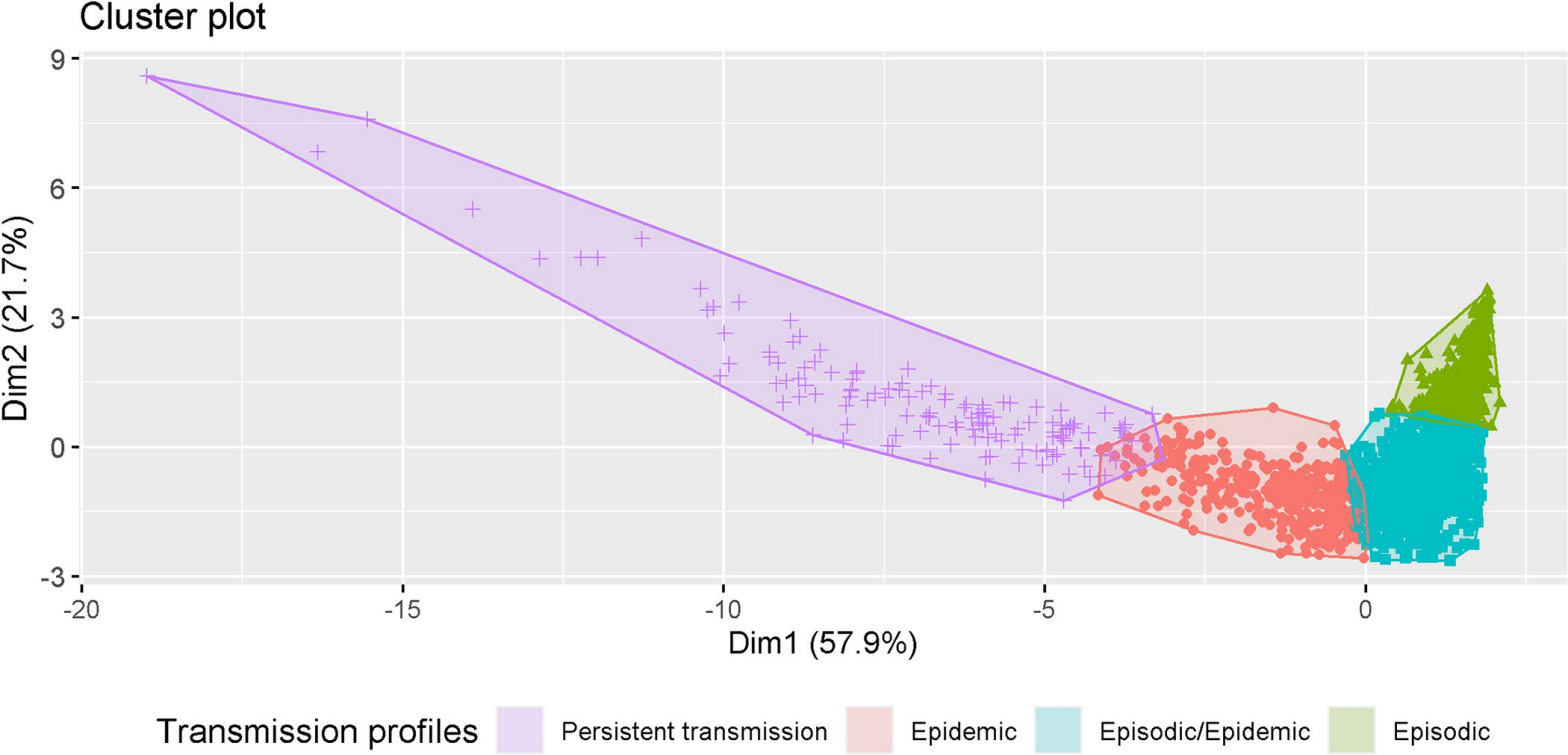
Cluster chart showing the four clusters of municipalities, each presenting different dengue transmission profiles.

Fig 5 presents the output of the elbow method, indicating that 3 to 5 clusters would be sufficient to classify the municipalities according to their epi-features. Subsequently, the PAM algorithm *(k-medoids*) was applied, considering k = 2, 3, 4, or 5 clusters. Fig 6 shows the distribution of the municipalities, represented by the points, forming an elongated cloud along the first principal component, without a clear separation (spacing) between them. The first two principal components explained 79.6% of the variation in the data, indicating that the epi-features have high descriptive power. By visual inspection, it is evident that the classification in 5 clusters produces significant overlaps between them. A classification in 3 or 4 clusters produces better-defined clusters.

**Fig 7:**
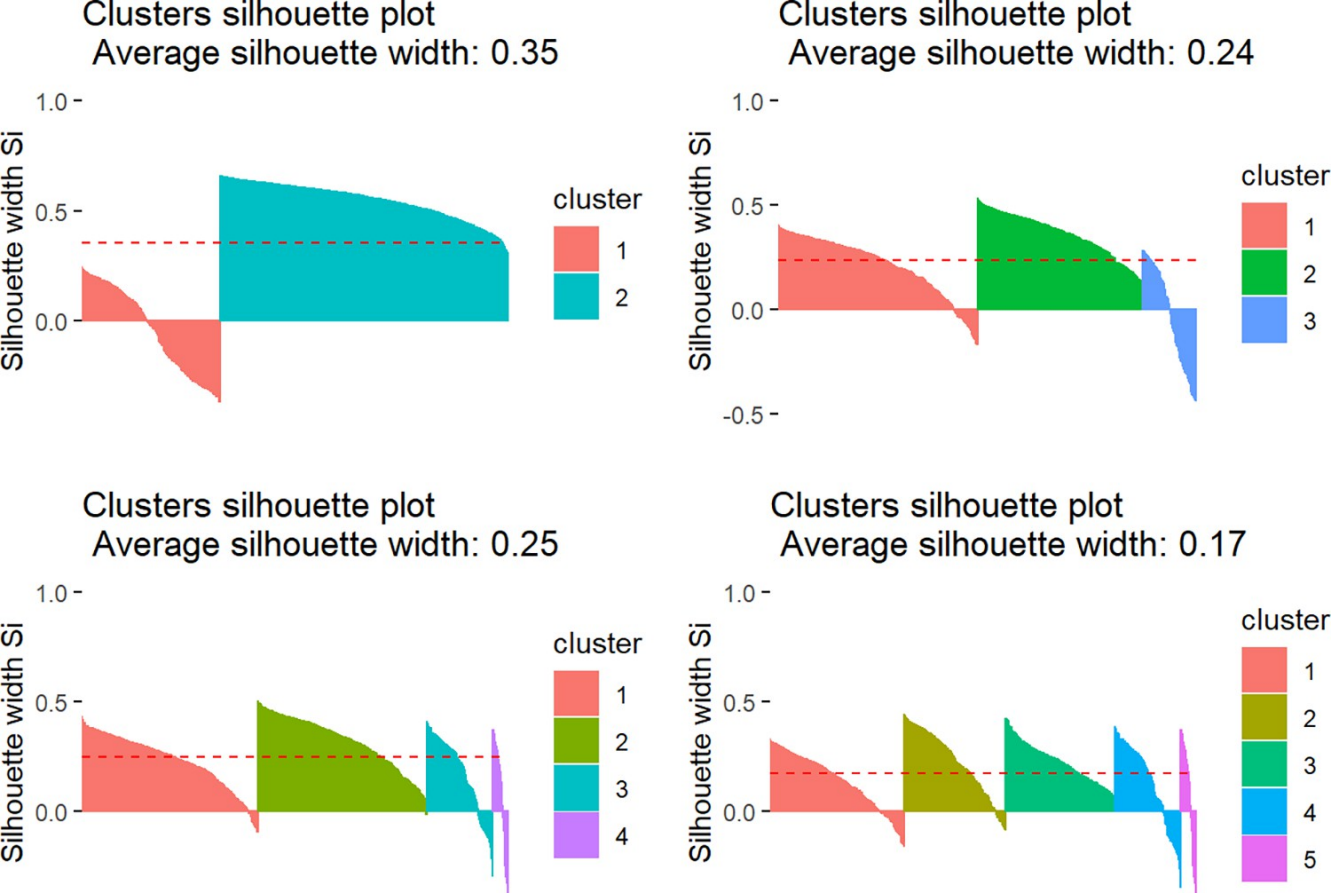
Silhouette graph showing four of groups with 2,3,4, and 5 clusters.

The silhouette method was used to assess the goodness of the classification in 2, 3, 4, or 5 groups (Fig 7). According to the average silhouette width, the best number of clusters should be k = 2, followed by k = 4 (the larger the silhouette value, the better). The graphs also show each municipality’s silhouette coefficients, which measure their degree of similarity to those in the cluster they were assigned to. High values greater than zero imply good matches, while values less than zero signify mismatches. With, k = 2, there is one well-defined group and one poorly defined group, with 50% of mismatches. With, k = 4, on the other hand, the first three clusters are well-defined, and only the fourth group is less defined. Between 3 and 4 clusters, the width measurement was marginally better for 4 clusters. With k = 4, we observe that cluster 2 has all the data (municipalities) well classified. Cluster 1 has only a small fraction of poorly classified municipalities. The quality of cluster 1 is better with k = 4 compared to k = 3 or k = 2.

As a result, we chose the classification in four groups, which also provides us with a more detailed description of the patterns found.

**Fig 8:**
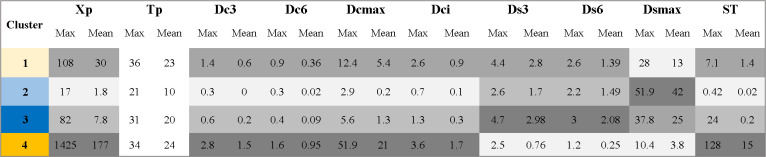
Summary statistics of epi-features within the four clusters. Average and maximum value of the epi-features. The rank of values is indicated by the degree of gray shade (higher values = darker gray).

Fig 8 shows summary statistics of the epi-features in each of the four clusters. Note that cluster 4 tended to concentrate the municipalities with the highest values ​​of Dc* epi-features, that is, the indicators of high disease incidence and persistence. Cluster 1 presented the second-highest values. Cluster 3, on the other hand, presented the highest values ​​of Ds* epi-features, which are indicators of intermittency or absence, while cluster 2 had low values ​​on all indicators except Dsmax. Based on these observations, it was possible to associate the four clusters with typical dengue transmission profiles, as described next:

### Persistent dengue transmission profile

Persistent dengue transmission is defined by medium to high frequency of weeks with disease occurrence, with few or no periods without records of cases, thus implying conditions for continuous maintenance of viral circulation. Cluster 4 was interpreted as Persistent Transmission because the municipalities in it presented large epidemic peaks (Xp) (mean = 177, maximum = 1,425), high frequency of periods with 3 weeks (mean = 2, 8 times/year) or 6 weeks (mean = 0.95 times/year) with dengue cases (Dc3 and Dc6), and the largest continuous periods with cases (Dcmax), with 51.9 of 52 weeks. Cluster 4 also presented the lowest frequency of consecutive weeks without cases (Ds3, Ds6). In this cluster, the ST epi-feature shows a trend component greater than the seasonality component, with an average of 15. A typical example of a municipality with persistent dengue transmission is Nova Friburgo in the mountainous region of Rio de Janeiro.

### The epidemic dengue transmission profile

Epidemic dengue transmission is defined by the seasonal or periodic occurrence of weeks with high incidence of cases, interspersed with periods with no cases. The transmission period can be short or long, but incidence in the transmission period can reach high values. After careful consideration of the distribution of the epi-features among the clusters, we assigned this profile to Cluster 1. In this cluster, municipalities presented epidemic peaks (Xp) with moderate values ​​(mean = 30, maximum = 108) and presented less frequent periods with 3 or 6 consecutive weeks with cases (Dc3 and Dc6), when compared to the persistent pattern. On average, Ds6 = 1, suggesting one period per year. Furthermore, these municipalities presented 3 or 6 weeks without cases (Ds3 and Ds6) only twice a year on average. The Dcmax, on average, was 5 consecutive weeks, corresponding to approximately two generations of dengue transmission. The maximum period without cases (Dsmax) was 13 weeks on average, accounting for about 25% of an epidemiological year. Finally, the ratio between trend and seasonality (ST) suggests that the seasonality of dengue in municipalities within this cluster is higher than those in cluster 4, having an average value of 1.4, reaching 7.1. This means that these municipalities have more seasonally marked periods of disease occurrence, an indicator of climate-mediated transmission. A typical example of a municipality with an epidemic dengue profile is Guaxupé, located in southwest Minas Gerais state.

### The episodic / epidemic dengue transmission profile

The Episodic / Epidemic transmission profile encompasses the transition between the Episodic dengue transmission profile (which will be described below) and the above-mentioned Epidemic profile. It is characterized by the occurrence of a few weeks per year with dengue cases and the non-sustainability of dengue transmission for a long period. When there are cases, the number exceeds those observed in the Episodic profile. Cluster 3 was assigned to this profile because the municipalities in it showed case peaks (Xp) with intermediate values ​​between the Episodic and Epidemic profiles (mean = 7.8, maximum = 82) and there was a low frequency, although higher than in the Episodic profile, of periods with 3 or 6 consecutive weeks with cases (Dc3 and Dc6 with a mean of 0.17 and 0.09 times/year, respectively). Cluster 3 contains municipalities with long periods without cases (Dsmax, on average, is 24.7 weeks (47.5%)). Finally, the ratio between trend and seasonality (ST) shows that the trend varies considerably in relation to the seasonality, with values ​​ranging from 0.2 to 2.4. An example of a municipality with an Episodic/Epidemic dengue profile is Confins, located in Greater Metropolitan Belo Horizonte, capital of Minas Gerais state. Confins is 39 km from the city of Belo Horizonte, classified in the Persistent Transmission dengue profile.

### The episodic dengue transmission profile

The Episodic profile stands out for an irregular occurrence of the disease in a particular location. When there are cases, they cannot be sustained, the cycle is short, and the number of cases is small. There are long periods during which the disease is not detected. Cluster 2 was interpreted as Episodic, as the municipalities in it presented the lowest dengue peaks (Xp), in fact almost non-existent (mean = 1.8, maximum = 17), there were rare periods with 3 or 6 consecutive weeks with cases (Dc3 and Dc6), about 0.02 times a year, and long case-free periods (Dsmax) of 41.7 weeks on average. The ratio between trend and seasonality (ST) of dengue in each location shows that the trend is smaller than the seasonality component. An example of a municipality with an Episodic dengue profile is Davinópolis, located in western Maranhão state. A summary of the characteristics of the transmission profiles can be seen in Table 2.

**Table 2:** Description of the four dengue transmission profiles that emerged from the cluster analysis of the dengue time series.

Transmission Profile	Features
**Episodic**	• Irregular and low-frequency occurrence of dengue in a certain location. • When there are cases, the disease is sustained for a short period. • Low incidence. • The period for an increase in the number of cases to occur again is long, and dengue may not be detected for some time, that is, cases occur occasionally.
**Episodic/Epidemic**	• The transitional profile between the Episodic and the Epidemic. • Occurrence of a few weekly cases. • Transmission is not sustained for long periods, but when cases occur, they exceed the expected limit for that location.
**Epidemic**	• Seasonal or periodic occurrence of high dengue incidence in a given location, interspersed with periods with absence of cases. • The sustaining period can be of short or long duration. • Incidence in the period remains high.
**Persistent Transmission**	• Regular and high dengue incidence in a specific location, with few or no periods with no cases. • Transmission sustained for long periods. • High incidence.

### Distribution of the dengue profiles among states

**Fig 9:**
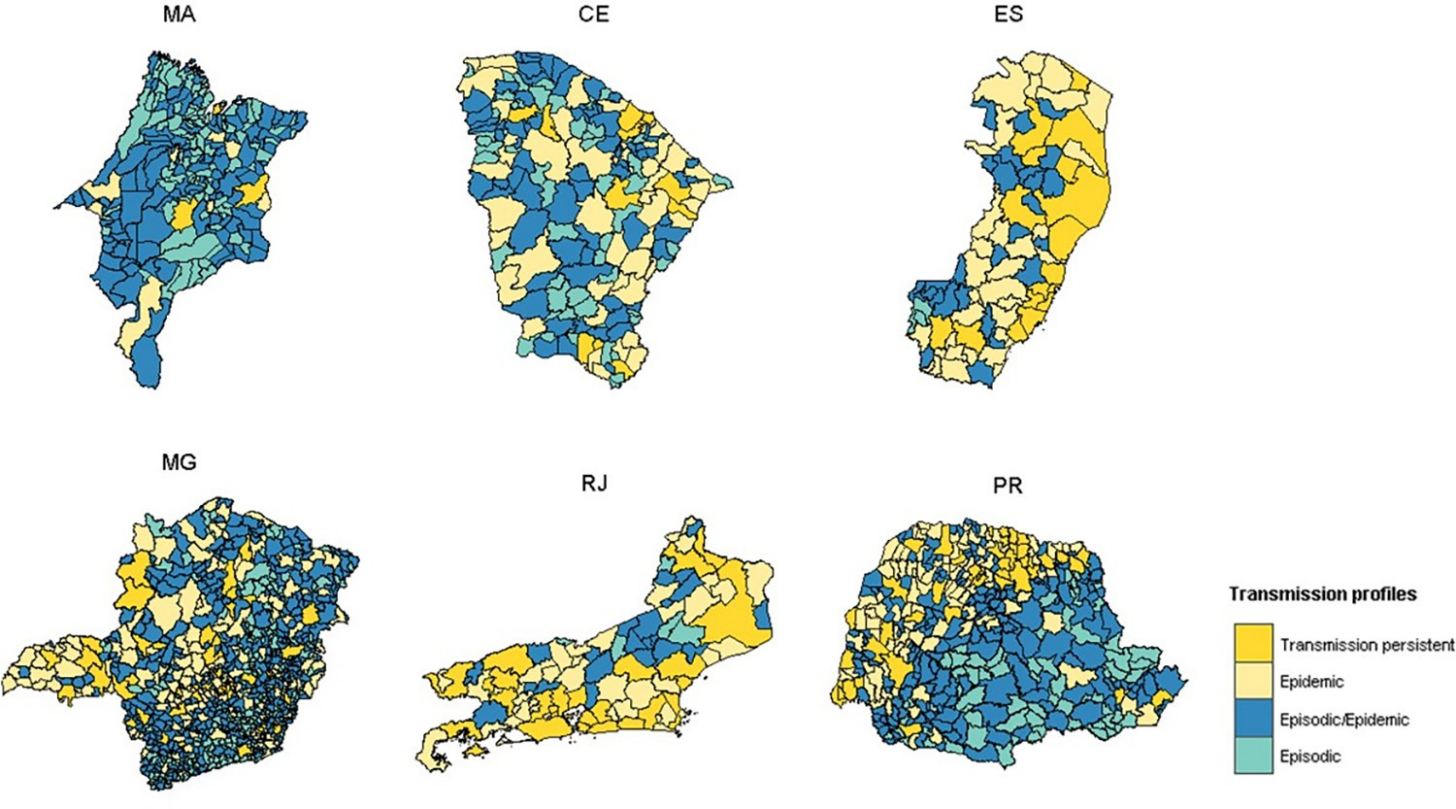
Maps of the four dengue transmission profiles in the Brazilian states. From north to south: Maranhão (MA), Ceará (CE), Espírito Santo (ES), Minas Gerais (MG), Rio de Janeiro (RJ), and Paraná (PR). Service Layer Credits: Sources: https://www.ibge.gov.br/geociencias/organizacao-do-territorio/malhas-territoriais/15774-malhas.html?=&t=downloads.

Of all 1,823 municipalities, only 7.8% were classified as having Persistent Dengue Transmission, compared to 21.3% with Epidemic Dengue Transmission, 43.2% with Episodic/Epidemic Dengue Transmission, and 27.6% with Episodic Transmission (Table 3). The Episodic and Episodic/Epidemic profiles were the most predominant types in Maranhão (94% of municipalities), Minas Gerais (75.8%), Ceará (68.5%), Paraná (63.4%). In Rio de Janeiro and Espírito Santo, the predominant profiles were Epidemic and Persistent Transmission, respectively, with 69.4% and 64.1% of the municipalities (Fig 9).

**Table 3:** The dengue transmission profiles of the six states. In relation to the proportion of municipalities, range of population size, and incidence (per 100,000 inhabitants).

Profile	Variables	States					
		MA	CE	ES	MG	RJ	PR
**Total municipalities**		217	184	78	853	92	399
**Episodic**	Municipalities (%)	101 (46.5)	55 (29.9)	3 (3.9)	271 (31.2)	5 (5.4)	68 (17)
	Population size (x1,000)	4.4–45	4.4–46	4.6–9.3	0.8–33	7.2–15	2.6–33
	Dengue Incidence	0–7.15	0.06–9.79	0.6–1.5	0–19	1.8–9	0–9
**Episodic/Epidemic**	Municipalities (%)	103 (47.5)	71 (38.6)	25 (32)	381 (44.6)	23 (25)	185 (46.4)
	Population size (x1,000)	3.3–120	3.6–74	8–29	1.4–135	5.4–174	1.4–234
	Dengue Incidence	0.19–31	0.17–27	1.06–18	0.34–49	1.13–44	0.16–79
**Epidemic**	Municipalities (%)	10 (4.6)	47 (25.6)	33 (42.3)	156 (18.3)	39 (42.3)	104 (26)
	Population size (x1,000)	46–253	11–126	5.8–45	3.7–165	12–298	1.8–341
	Dengue Incidence	1.02–10	1.22–44	4.5–52	1.4–67	1.9–38	0.6–95
**Persistent Transmission**	Municipalities (%)	3 (1.4)	11 (5.9)	17 (21.8)	45 (5.3)	25 (27.1)	42 (10.5)
	Population size (x1,000)	86–1,000	48–2,600	26–4,900	24–2,600	28–6,700	5–1,800
	Dengue Incidence	4–24	4.5–21	13.9–41	8.15–57	2.4–152	2.24–153

Table 3 shows the range of population sizes of the municipalities classified according to each dengue transmission profile. In general, episodic transmission is found in less populated municipalities, while epidemic and endemic transmissions are found in the most populated ones. In Ceará and Maranhão, the Episodic Dengue profile is only found in municipalities with 4,000 to 47,000 inhabitants. This population range differs considerably from those with the Persistent Transmission profile, only present in municipalities with more than 48,000 inhabitants, in Ceará, or 86,000, in Maranhão. Municipalities with Epidemic dengue profiles start with 11,000 inhabitants in Ceará and 47,000 in Maranhão.

In the states of Espírito Santo (ES), Minas Gerais (MG), and Rio de Janeiro (RJ), located in the more populated and urbanized Southwest region of Brazil, we found the Persistent Transmission profile in municipalities with populations greater than 25,000. Meanwhile, Episodic Transmission occurs in municipalities with fewer than 9,800 inhabitants in ES, 15,000 in RJ, and 30,000 in MG. The Epidemic profile, on the other hand, can be detected in very small municipalities in ES and MG, with as few as 3,700 and 5,800 inhabitants, respectively. Paraná, located in the subtropical region, showed the largest differences in population size per profile when compared to other states, especially in the Persistent Transmission profile. Ten municipalities in northern Paraná, in Greater Metropolitan Londrina, presented Persistent Transmission despite having fewer than 20,000 inhabitants. In this state, some municipalities with fewer than 2,000 inhabitants showed Epidemic profiles.

Table 3 also shows the range of mean annual dengue incidence in the municipalities classified in each cluster in each state. In the municipalities with an Episodic Transmission profile, mean dengue incidence varied from 0 (Paraná) to 19 (Minas Gerais) cases per 100,000 inhabitants. In the Episodic/Epidemic profile, dengue incidence varied from 1.6 (Ceará, Maranhão, and Paraná) to 1.1 (in Rio de Janeiro and Espírito Santo). The Epidemic dengue profile was found in municipalities with dengue incidence varying from 1.8 cases per 100,000 inhabitants in Paraná to 50–90 cases per 100,000 inhabitants in all states. Finally, Persistent Transmission appeared in municipalities ranging from 2 to 153 cases per 100,000 inhabitants. Although the dengue incidence intervals of the four profiles overlap, the higher the incidence, the greater the tendency of municipalities to have Epidemic or Persistent Transmission.

## Discussion

This study aimed to propose a classification of dengue transmission profiles based on epi-features extracted from the time series of cases in Brazilian municipalities varying in population size, mean annual dengue incidence, and climate. The Persistent Transmission profile predominated in the states of Espírito Santo and Rio de Janeiro, characterized by tropical humid and warm climate, higher population density, and more urbanization, while the Episodic and Episodic/Epidemic profiles were concentrated in Ceará and parts of Maranhão and Minas Gerais, where the climate is predominantly dry, and the municipalities are less populated and more rural. Paraná, in the subtropical region, showed a clear spatial separation of profiles, likely associated with the temperature limits for dengue transmission.

Climate and population density are important drivers of dengue transmission. All large municipalities in this study (greater than 350,000 inhabitants) presented Persistent Transmission, independent of climate. Meanwhile, the Episodic profile was limited to municipalities with fewer than 50,000 inhabitants. In Peru, a study at the state level by Chowell *et al*. (2009) [44] found a critical community size (CCS) of 350,000 inhabitants for dengue persistence. Despite the similar number, there are important differences between these studies. In our analysis, the probability of persistence increases with population size, although there is Persistent Transmission in small municipalities starting with 25,000 inhabitants, as seen in Table 3. Special attention should be devoted to these municipalities capable of maintaining Persistent Transmission even with relatively small populations. Dengue transmission requires a pool of susceptible humans and a large and competent mosquito population [24,45,46]. Mosquito population abundance may correlate with human density since the dengue vector is an urban and domestic species [10,14,15,19]. However, other factors can modify this association, such as the availability of breeding sites and a favorable climate [21,47–49]. This multiplicity of factors may explain the wide range of population sizes associated with the Episodic/Epidemic and Epidemic profiles.

Several studies have shown the association between climatic variables and dengue transmission [17,21,24,50,51]. In Ceará, the climate characterized by extended dry seasons may explain the presence of few municipalities with Persistent Transmission profiles, because low humidity and very high temperatures (on average 30°C) negatively influence the vector’s biological cycle. Liang *et al*. (2019) and Seah *et al*. (2020) [52,53] show that extremely high temperatures can prevent the development of the *Aedes* mosquito. Meanwhile, Costa *et al*. (2013) and Souza *et al*. (2018) [54,55] discuss that water shortages can lead to the habit of accumulating water for consumption, contributing to the maintenance of *Aedes* populations even in unfavorable climates.

Paraná is situated in the subtropical zone. The state has two climates, humid subtropical, where there are more municipalities with Persistent and Epidemic profiles. A cluster of municipalities with the persistent transmission is found in the state, including the city of Londrina and its surroundings. This case deserves closer scrutiny, as this region has shown Persistent Transmission even in small municipalities with 2,400 inhabitants. This phenomenon may be explained by the high connectivity between these populations, maintaining the disease in a source-sink dynamics. In southern Paraná, with mean temperatures between 10 and 15°C, there are mainly municipalities classified as having Episodic and Episodic/Epidemic Transmission. In the literature, other authors have defined transmission profiles (“epidemic types”) of transmissible diseases based on population size and associated variables for other transmissible diseases. Among the seminal studies, Bartlett (1960) [56] used a set of methods including deterministic and stochastic models to classify the dynamics of measles transmission according to its occurrence, population size, and periodicity, deriving three profiles: type I epidemics, which occur in places with large urban populations, where measles is endemic with periodic outbreaks without extinction of cases; type II epidemics, where there are regular epidemic outbreaks and periods of non-transmission; and type III epidemics, often in small populations that do not have the minimum necessary size to sustain the disease, with irregular transmission behaviors, without well-defined seasonality. Further work based on this classification using mathematical modeling elucidated the spatial dynamics of measles as a wave travelling from large municipalities to smaller ones, in source-sink dynamics [57]. In our study, the dengue transmission profiles are analogous to Bartlett’s epidemic types, despite the different methodologies: type I is similar to Persistent transmission, type II is similar to Epidemic, and type III is similar to the combination of Episodic and Episodic/Epidemic profiles. Future work should investigate the spatial distribution of these profiles and the possible mechanisms explaining these patterns.

The usefulness of classifying epidemic profiles for defining public health policies related to dengue is exemplified by the World Health Organization manual (2013) that categorizes Southeast Asian countries according to their epidemiological characteristics for dengue. Four classes were established: Class A comprises countries with hospitalizations and deaths of children from the disease and circulation of the virus in rural areas while dengue is endemic in urban areas; Class B refers to countries with the occurrence of frequent epidemic cycles accompanied by expansion into the interior; Class C presents indefinite disease endemicity, and Class D has no evidence of endemicity. Each of these classes emerges from a combination of profiles, which could be determined in a more systematic way by using the methodology introduced here. With this bottom-up approach, the municipal classification allows building a new classification at the state or national level.

There are other studies on dengue transmission patterns and their determinants in Brazil, using different methodologies. Silva *et al*. (2020)[58] used a multivariate method to identify, among the capitals of Northeast Brazil, the level of similarity between dengue and climatic, sociodemographic, and sanitation variables between 2001 and 2012. Lowe *et al*. (2021) [59] used spatio-temporal modeling to investigate the delayed and nonlinear effects of extremely wet and extremely dry conditions on dengue risk across Brazil, with six different biomes, and concluded that environmental factors such as drought and rainfall influence dengue transmission patterns.

There are limitations to the present analysis. First, differences in the dengue profile can be influenced by the reporting effort, for example, transmission can occur even if there are no reported cases. Second, in Brazil, chikungunya emerged in late 2014, causing two epidemic years, 2015 and 2016, and Zika emerged in 2015, with two epidemic years, 2016 and 2017. In these years, the number of dengue cases may have been affected by misdiagnosis. However, in the other seven years of the study’s time series (2010–2019) dengue was always dominant, corroborating the fact that Brazil is endemic for the disease. Third, the proposed epi-features represent only a subset of all possible descriptors that we could derive from these time series. Other characteristics may provide a better discrimination between Clusters 3 and 4. Fourth, the classification may change if the study includes more municipalities or considers a longer time series. Finally, the classification proposed in this study used the PAM algorithm method, although there are studies that describe other clustering methods that could be used, such as the Kamila algorithm and Latent Class Analysis, for example [60], the one used met all the objectives of the study by some factors. PAM is an algorithm that can previously select the number of clusters, which was useful since the hypothesis of the study was the existence of at least 3 groups of dengue profiles. Another factor is the way the algorithm works with discrepant data, which was essential since some municipalities had a large number of cases.

## Conclusions

The classification of dengue transmission profiles based on the epi-features of the epidemic curves provided a useful characterization. A gradient of profiles was obtained, ranging from Episodic to Persistent Transmission. We found evidence of an association between these profiles, climate and population size, consistent with the literature on dengue. All the proposed epi-features were important for the classification of municipalities in relation to their dengue transmission, but there is no limit to the inclusion of other descriptors in futures to increase the discriminatory power between the clusters. This method can also be adapted to other communicable diseases, such as other arboviruses and respiratory diseases, as the descriptors are easily modifiable to measure the epidemic cycle of other acute diseases.

In conclusion, the classification of municipalities in temporal transmission profiles can bring significant benefits for the effective monitoring of the disease. It can be used to improve dengue alerts, such as those carried out by Infodengue, as well as to develop more precise public policies to combat this disease.

## Supporting information

S1 Fig Indication matrix for epi-features. (A) Matrix with the 13 initially proposed epi-features; (B) Matrix with 10 epi-features after excluding Dsmed, Dcmed and P.
(TIF)
