# Supplementary figures and images for: How heterogeneous is the dengue transmission profile in Brazil? A study in six Brazilian states
Source: PLoS Negl Trop Dis. 2022 Sep 12;16(9):e0010746. doi: 10.1371/journal.pntd.0010746 (PMC9499305; doi:10.1371/journal.pntd.0010746)

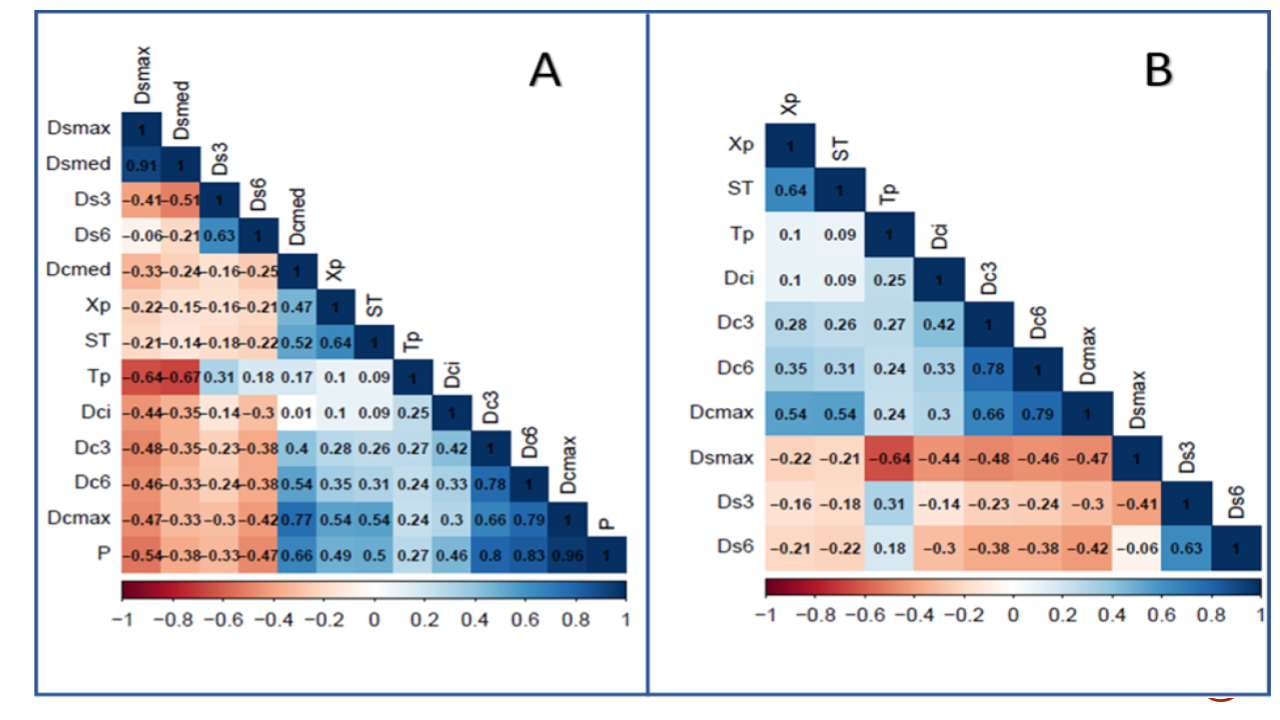

Supplement: S1 Fig — (A) Matrix with the 13 initially proposed epi-features; (B) Matrix with 10 epi-features after excluding Dsmed, Dcmed and P. (TIF) [file pntd.0010746.s001.tif]
